# Clinical and Epidemiological Characteristics of Hospitalized COVID-19 Patients in an Isolation Centre in South-West Nigeria

**DOI:** 10.7759/cureus.46992

**Published:** 2023-10-13

**Authors:** Samuel A Dada, Bolade F Dele-Ojo, Taiwo H Raimi, Peter Ojo, Adekunle O Adeoti, Joseph O Fadare, Mojeed O Rafiu, Oluwamayowa E Dada, Jimoh K Olabanji

**Affiliations:** 1 Department of Medicine, Ekiti State University Teaching Hospital, Ado Ekiti, NGA; 2 Department of Internal Medicine, Institute of Viral Haemorrhagic Fever and Emergent Pathogens, Irrua Specialist Teaching Hospital, Irrua Edo State, NGA; 3 Department of Health Information Management, Federal Teaching Hospital, Ido Ekiti, NGA; 4 Department of Surgery, Ekiti State University Teaching Hospital, Ado Ekiti, NGA

**Keywords:** clinical features, nigeria, mortality rate, epidemiology and public health, covid-19

## Abstract

Introduction: The clinical presentation of coronavirus disease 2019 (COVID-19) can vary widely, and while the primary infection involves the respiratory system, other organs can also be affected. This study presents the clinical and epidemiological characteristics of hospitalized COVID-19 patients in a tertiary hospital in Ado Ekiti, South-West Nigeria.

Materials and methods: This is a retrospective study involving COVID-19 patients admitted to the isolation ward between August 2020 and January 2021. The data used for this study was obtained from the patient’s medical record, which includes demographic characteristics, clinical presentation, baseline co-morbidities, and laboratory investigations.

Results: The average age of the patients was 60.3 years, and more than two-thirds were male. The most common symptoms were fever, shortness of breath, cough, and tiredness. Comorbidities identified among the patients included diabetes mellitus, heart disease, obesity, and chronic kidney disease. The most common radiological findings were bilateral homogeneous patchy opacities and peripheral fluffy infiltrates. The overall mortality rate was 21.9%, with 13 deaths in patients with severe disease. Age and duration of admission were found to be significant predictors of death.

Conclusion: The results of this study provide valuable insights into the clinical presentation of COVID-19 in Nigeria and may guide future management strategies for similar infections.

## Introduction

In December 2019, the coronavirus disease 2019 (COVID-19) pandemic was declared as a result of the emergence of severe acute respiratory syndrome coronavirus-2 (SARS-CoV-2), which originated in Wuhan, China, and rapidly spread worldwide by March 2020 [1,2]. This was later designated coronavirus disease 2019 (COVID-19) in February 2020 by the World Health Organisation (WHO). As of September 2023, Nigeria has had a total of 266,675 confirmed cases of COVID-19 with mortality above three thousand [3].

The clinical spectrum of COVID-19 varies widely, while the primary infection targets the respiratory system, other organ systems such as the renal, haematological, neurological, gastrointestinal and endocrine systems are affected. The affectation can be asymptomatic, mild to moderate or life-threatening complications, including acute respiratory distress syndrome (ARDS) or death.

Symptomatic patients with COVID-19 may present with fever in about 85% of cases during their illness course [4]. Cough with or without sputum production including dyspnea, sore throat, and nasal congestion may be present. Constitutional symptoms such as muscle or bone aches, chills and headache are common while some patients have been reported to present with loss of taste and smell. Shock, acute respiratory distress syndrome (ARDS), acute cardiac injury, acute kidney injury (AKI), and deaths were reported in severe cases [4,5]. COVID-19 disease has shown distinct symptomatology as well as outcomes across geographical borders owing to multiple factors such as population distribution, genetic make-up, baseline diseases, immune constitution, health infrastructure and probably some factors that are yet to be completely understood [6]. The synthesis and review of presenting symptoms and clinical features of COVID-19 infections may lead to a better understanding of the disease process in the Nigerian context. This information may be valuable in the management of similar infections in the future. We present hospitalized COVID-19 patients in our isolation centre, a tertiary hospital in Ado Ekiti, South West Nigeria.

## Materials and methods

This is a retrospective and descriptive study of the clinical and epidemiological characteristics of hospitalized COVID-19 patients in our isolation ward between August 2020 and January 2021. The treatment centre provided medical care and closely monitored the patients. Ethical Review Committee at Ekiti State University Teaching Hospital (EKSUTH), Ado Ekiti, Nigeria issued approval for the study with an approval number EKSUTH/A67/2020/01/016.

Study location

The isolation centre is located at Ekiti State University Teaching Hospital (EKSUTH) in Ado Ekiti. Ado Ekiti, a state capital city in Nigeria, is situated in the South-Western region. The city has an urban character and is home to a diverse population. As of the latest available data, the estimated population of Ado Ekiti stands at approximately 366,280. This marks a notable increase from the 2006 population figure of 308,621, indicating the city's ongoing growth and development.

Data collection and handling

The data used for this study was obtained from the patient’s medical records which include the demographic characteristics, clinical presentation, baseline co‐morbidities and laboratory investigations. The data were double-checked by two medical officers after entering into the datasheet. Our inclusion criteria were adult patients with COVID-19 confirmed by polymerase chain reaction (PCR) testing who were admitted at the EKSUTH isolation centre during the study period. The exclusion criteria were patients who tested positive for COVID-19 and who were still on admission at the end of January 2021 and patients with incomplete medical records. Permission to conduct the research was obtained from the ethical review committee of the hospital.

Definition of terms

Fever was defined as a recorded temperature of 37.5°C or higher while hypoxia was defined as an oxygen saturation <94% on ambient air, or a requirement of supplemental O2 [7]. Body mass index in kg/m^2^ was calculated as a person’s weight in kilograms divided by the square of the height in meters and categorized as underweight (< 18.5 kg/m^2^), normal weight (18.5 to 24.9 kg/m^2^), overweight (25.0 to 29.9 kg/m^2^) and (≥ 30.0 kg/m^2^) obese.

The primary presenting symptom encompassed a singular symptom or a cluster of symptoms that the patient had during the initial assessment, including manifestations such as fever, myalgia, cough, and dyspnoea. This symptomatology was subsequently classified into distinct categories of asymptomatic and symptomatic presentations depending on whether they were present or not.

COVID-19 was classified as a mild, moderate and severe disease [7,8]. Mild disease includes symptomatic patients without evidence of viral pneumonia or hypoxia. Moderate diseases are patients with clinical and radiological signs of pneumonia but without signs of severe lung involvement. Severe disease was defined as a confirmed patient with the above symptoms in addition to extensive lung involvement, often with bilateral lung infiltrates and respiratory rate > 30 breaths per minute, severe respiratory distress and SpO2 < 90% in room air. The severity was categorized as not severe (asymptomatic, mild, and moderate) and severe disease. 

Comorbidity, in this context, refers to the concurrent presence of one or multiple underlying health conditions or diseases such as diabetes mellitus, systemic hypertension, obesity, kidney disease, cancer, sickle cell anaemia etc., alongside the COVID-19 infection.

Statistical analysis

Statistical analyses were conducted using SPSS version 23.0 (IBM Corp., Armonk, NY). Continuous variables were expressed as mean ± SD or median (IQR), depending on their distribution. Categorical variables were compared between deceased and discharged patients using the Chi-square test and continuous variables by using the Student t-test or Mann-Whitney U test as appropriate. Bivariable logistic regression was used to assess the relative impact of predictor variables on the outcome (discharge or death) while controlling for potential confounding factors. Multiple variables were adjusted in the logistic regression models. Results were presented with tables. A p-value of ≤0.05 was considered to be statistically significant.

## Results

Sixty-four confirmed cases of COVID-19 were enrolled in this study. The mean age of the patients was 60.30 ± 17.28 years, with a range of 23 to 89 years. Male gender accounted for more than two-thirds of the patient population (Table 1).

**Table 1:** Comparison of clinical and radiological characteristics of patients

Variables	All patients n=64	Discharged n=50	Deceased n=14	X2-chisquare
Gender				
Male	44(68.8)	32(72.7)	12(27.3)	
Female	20(31.3)	18(90.0)	2(10.0)	0.112
Shortness of breath				
No	9(14.1)	8(88.9)	1(11.1)	
Yes	55(85.9)	42(76.4)	13(23.6)	0.399
Fever				
No	8(12.5)	7(87.5)	1(12.5)	
Yes	55(87.5)	43(76.8)	13(23.6)	0.479
Muscle ache				
No	36(56.3)	28(77.8)	8(22.2)	
Yes	28(43.8)	22(78.6)	6(21.4)	0.939
Sore throat				
No	61(95.3)	47(77.0)	14(23.3)	
Yes	3(4.7)	3(6.0)	0	0.348
Loss of smell				
No	60(93.8)	47(78.3)	13(21.7)	
Yes	4(6.3)	3(75.0)	1(25.0)	0.876
Loss of taste				
No	57(89.1)	44(77.2)	13(23.8)	
Yes	7(10.1)	6(85.7)	1(14.3)	0.607
Headache				
No	60(93.8)	47(78.3)	13(21.7)	
Yes	4(6.3)	3(75.0)	1(25.0)	0.89
Chest pain				
No	54(84.4)	41(75.9)	13(24.1)	
Yes	10(15.6)	9(90.0)	1(10.0)	0.323
Vomiting				
No	60(93.8)	46(76.7)	14(23.3)	
Yes	4(6.3)	4(100.0)	0	0.274
Tiredness				
No	14(21.9)	11(78.6)	3(21.4)	
Yes	50(78.1)	39(78.0)	11(22.0)	0.964
Diarrhoea				
No	63(98.4)	49(77.8)	14(22.2)	
Yes	1(1.6)	1(100.0)	0	0.594
Confusion				
No	57(89.1)	46(80.7)	11(19.3)	
Yes	7(10.9)	4(57.1)	3(42.9)	0.155
Chest X-ray findings				
Normal	20(31.3)	19(95.0)	1(5.0)	
Abnormal	44(68.8)	31(70.5)	13(29.5)	0.028

The occupation and socio-demographic characteristic of the patients is shown in Table 2, with retirees constituting the majority (26.6%), while medical personnel accounted for about 10%. The majority of the patients are well-educated, with over 90% having completed some form of post-secondary school education. Similarly, a greater proportion were married (90.6%).

**Table 2:** Occupation distribution of the patients a= farmers, self-employed, employed in non-formal sector

Occupation	Frequency (N%)
Retiree	17(26.6)
Trader	14(21.9)
Lecturer &Student	8(12.5)
Medical Personnel	6(9.4)
Government worker	6(9.4)
Politician	3(4.7)
Artisan	2(23.1)
Others^a^	8(12.5)

The duration of symptoms before diagnosis ranged between one and 21 days, with a median of seven days. The overall mortality was 21.9%, of which patients with severe disease 58(90.6%) accounted for 20.3%. In the majority of cases (71.7%), the radiological findings of the patients showed bilateral homogeneous patchy opacities and peripheral fluffy infiltrates. Other minor findings included pleural effusion and cardiomegaly. However, no chest CT scan was performed due to logistic and financial constraints. Co-morbidities identified among the patients include hypertension (59%) diabetes mellitus (48.4%), heart-related disease (10.9%), obesity (15.6%), and chronic kidney disease (6.3%) (Table 3).

**Table 3:** Comorbidity condition among the patients CKD=chronic kidney disease

Comorbid	All patients N(%)	Discharged N(%)	Deceased N(%)	Chi-square
Diabetes				
Yes	31(48.4)	21(32.8)	10(15.6)	
No	33(51.6)	29(45.3)	4(6.3)	0.051
Heart diseases				
Yes	7(10.9)	6(9.4)	1(1.6)	
No	57(89.1)	44(68.8)	13(20.3)	0.607
CKD				
Yes	4(6.3)	2(3.1)	2(3.1)	
No	60(93.8)	48(75.0)	12(18.8)	0.160
Obesity				
Yes	10(15.6)	6(9.4)	4(6.3)	
No	54(84.4)	44(68.8)	10(15.6)	0.131
Cancer				
Yes	1(1.6)	0(0.0)	1(1.6)	
No	63(98.4)	50(78.1)	13(20.3)	0.057
Hypertension				
Yes	38(59.4)	30(46.9)	8(12.5)	
No	26(40.6)	20(31.3)	6(9.4)	0.847

Compared with patients without dyspnoea, a significantly higher proportion of patients who experienced shortness of breath did not survive (20.3% vs 1.6%, χ² = 6.668, p = 0.036). As shown in Table 4, there was also a significant difference in the mean values of respiratory rate, C-reactive protein, serum creatinine, and white blood cell count between the deceased and discharged patients.

**Table 4:** Clinical and laboratory parameters between discharged and deceased patients BP=blood pressure, WBC=white blood cell, CRP=C-reactive protein. Values in mean ±SD

Variables	All patients	Discharged	Deceased	p-value
Age (years)	60.30±17.25	60±17.13	73.50±9.9356	0.001
Respiratory rate (Cycle/min)	33.48±5.54	32.56±4.79	36.79±6.89	0.038
Systolic BP (mmHg)	132.28±18.33	132.24±18.30	128.86±18.80	0.434
Diastolic BP (mmHg)	80.70±13.22	81.46±13.77	78.00±11.06	0.337
WBC (x10^9^cells/L)	8.61±5.47	7.12±3.94	13.72±7.09	0.005
Plasma glucose (mmo/L)	9.78±7.20	9.09±6.85	12.26±8.12	0.352
CRP (mg/dl)	73.56±52.52	59.62±45.44	123.36±46.59	0.001
PCV(%)	37.47±5.87	35.36±7.44	38.07±4.88	0.217
BMI (Kg/m^2^)	28.37±4.92	28.41±2.25	28.36±5.46	0.963
Creatinine (µmol/L)	109.78±64.58	96.63±44.15	156.70±99.14	0.011
Duration on admission(days)	8.05±3.90	8.80±3.74	5.36±3.32	0.003
SPO_2_ at admission (%)	85.45±12.00	89.38±6.34	71.43± 16.58	0.001

A logistic regression analysis was performed to evaluate the predictors of mortality among the patients (Table 5). The logistic regression model was statistically significant, χ2(5) = 30.768, p < 0.0005. The model explained 59.1% (Nagelkerke R²) of the variance in survival and correctly classified 77.8% of cases. Age and duration of admission were found to be significant predictors of death among the patients.

**Table 5:** Logistic regression assessing the predictors of mortality among the patients Variable(s) entered on step 1: gender, age, comorbidity, duration of admission, respiratory rate at admission.

Variables	B	Wald	df	Sig.	Exp(B)	95% C.I.
Gender (1)	.875	.757	1	.384	2.398	.334 -17.202
Age	.090	4.958	1	.026	1.095	1.011-1.185
Comorbidity (1)	1.148	1.488	1	.223	3.151	.498-19.920
Duration of admission	-.419	6.067	1	.014	.658	.471-.918
Respiratory rate at admission	.111	2.399	1	.121	1.118	.971 -1.287
Constant	-9.445	6.481	1	.011	.000	

## Discussion

The clinical spectrum of COVID-19 varies from asymptomatic infection or mild upper respiratory tract illness to severe interstitial pneumonia with respiratory failure and death. Studies have shown that the prominent symptoms of this viral infection include fever or chills, cough, dyspnoea, fatigue, myalgia, loss of taste or smell, sore throat, runny nose, nausea or vomiting, diarrhoea, and in some cases, other organs and systems are also affected. These symptomatologies were reflected in our patients. We found that the average age was 60.3 years, and most patients were male. The most common symptoms were fever, shortness of breath, cough, and tiredness. Co-morbidities identified among our patient included hypertension and diabetes mellitus among others. The most common radiological findings were bilateral homogeneous patchy opacities and peripheral fluffy infiltrates. The overall mortality rate was 21.9%, with 13 deaths in patients with severe disease. Age and duration of admission were found to be significant predictors of death.

In this study, more than two-thirds of the patients affected were male. This is similar to the study done in Thailand [9], Singapore [10], and China [11] but in contrast to other studies that reported more female prevalence [12,13]. Our retrospective study corroborates this observation and is consistent with findings from previous reports [14,15]. It has been documented that SARS-CoV-2 has a male bias in severity and mortality, which is consistent with previous coronavirus pandemics such as SARS-CoV and Middle East Respiratory Syndrome Coronavirus (MERS-CoV) [16]. This gender disparity further underscores the complex interplay of biological, immunological, and socio-demographic factors influencing COVID-19 outcomes [17].

In this study, the most common presenting symptoms were fever (87.5%), shortness of breath (87.3%), cough (79.7%), and tiredness (78.1%). The high prevalence of fever was similar to reports from other studies [18,19] but contrary to some previous studies that reported that fatigue, sore throat, shortness of breath and rhinorrhoea were the most common presenting symptoms [12,13,20].

The prevalence of loss of smell was low in our study, at only 6.3%. This is similar to the report of Pouw et al. (6.4%) [21] but in contrast to other studies that reported a high prevalence of anosmia (37.5%) [22]. Loss of smell and taste have been reported to be frequent symptoms among patients from Europe and the US more than among patients from China during the early pandemic [23].

Clinically, the progression of COVID-19 can be unpredictable. Some individuals may experience rapid deterioration from mild to severe symptoms, while others may remain asymptomatic or have only very mild symptoms throughout the course of their infection. For instance, loss of taste and smell are early symptoms of COVID-19 that may not be present in all cases [23].

The duration of symptoms before the diagnosis can vary depending on the type and severity of symptoms, the availability and accessibility of testing, and the individual's health status and behaviour. The median duration of symptoms before diagnosis in our study was seven days, this is within the normal range of incubation period of 2 to 14 days for COVID-19. However, some patients may have longer or shorter incubation periods, or may not develop any symptoms at all [24].

Confusion, headache, vomiting, and diarrhoea were documented in our study. These symptoms may mimic other diseases, but they can also be symptoms of COVID-19. Chest pain and muscle aches were also documented among our patients, similar to other reports. These symptoms are more likely to be related to COVID-19 if they occur with other respiratory symptoms.

The chest X-ray findings among our patients are similar to reports from other studies [10,25,26]. Patients who exhibited symptoms of COVID-19 typically experienced pronounced lung inflammation, which often led to the progression of the disease. Among these symptomatic patients, there was an increased likelihood of developing bilateral pneumonia and a decreased probability of pneumonia improvement compared to asymptomatic patients [25].

Overall, we recorded a mortality of 21.9% among the patients. A high mortality rate was observed among those who presented with initial symptoms of shortness of breath, in contrast to the report of Adekanmbi et al. [14] who reported a low death rate among patients with shortness of breath. Shortness of breath may be subjective hence it may not represent the actual severity of patients. A considerably higher number of patients experiencing shortness of breath did not survive compared to those who were discharged; 13 (20.3%) versus 1 (1.6%), χ² (2) = 6.668, p = 0.036.

We found a higher mortality rate of 17.2% among the elderly patients compared to 4.7% in the middle-aged patients (p = 0.014). In this study, 51.6% of the patients had one or more comorbid diseases. This was lower than the findings of Adekanmbi et al. (89.5%), who conducted a study in the same geographical region of the country [14], and a US study (55%) [27]. However, it was higher than the findings of Alasia et al. (32.8%)[28] and other studies conducted in Singapore 28.3% [10], China 15.8%[25] and Myanmar 37.8% [12]. This difference might be due to variations in the occurrence of chronic illnesses based on age, gender distribution, and geographical location.

Similar to the report of Adekanmbi et al. [14], hypertension (59.4%) was the most common underlying comorbidity in our patients. Other comorbidities included heart-related disease, obesity, and chronic kidney disease. Studies have demonstrated that older age, the presence of underlying diseases, and presenting symptoms such as shortness of breath and diarrhoea are poor prognostic indicators in hospitalized COVID-19 patients [14,29]. 

Limitations

As with any observational descriptive study that uses pre-existing data which may not be sufficient enough to identify predictors of mortality but rather a comparison of variables among the discharged or deceased patient due to a risk of incomplete documentation or misclassification of baseline characteristics. Additionally, the study's small sample size and single-centre design limit the generalizability of its findings.

## Conclusions

This retrospective study sheds light on the distinct characteristics and clinical outcomes of COVID-19 cases in our isolated centre in an urban city in South-West Nigeria. The findings underscore the multifaceted interplay of age, sex, presenting symptoms and co-morbidity in influencing the progression and outcome of COVID-19 cases. By delving into the local context, this study contributes to a more comprehensive understanding of the virus's impact on the Nigerian population. A comparison of our results with studies conducted globally reveals both similarities and differences. While age, presenting symptoms and comorbidity emerge as consistent determinants of COVID-19 severity across various populations, the intricate interaction of these factors with gender varies in its effect. These findings highlight the importance of considering the unique demographic and epidemiological factors of each region to tailor effective public health strategies and clinical interventions.

As the world continues to grapple with the COVID-19 pandemic and other emerging infectious diseases, collaborative research efforts across diverse populations remain pivotal. Our study underscores the significance of integrating local data into the global discourse to enrich our collective understanding of the disease. This research emphasizes the need for targeted interventions aimed at vulnerable subgroups, including the elderly and individuals with pre-existing health conditions. It is imperative that we continue to explore and analyse the multifactorial determinants of COVID-19 outcomes across diverse populations. Through such concerted efforts, we can advance our knowledge, refine our strategies, and ultimately work towards a healthier and more resilient world.
